# Antimicrobial use in food animals and human health: time to implement ‘One Health’ approach

**DOI:** 10.1186/s13756-020-00847-x

**Published:** 2020-11-07

**Authors:** Sunil Pokharel, Priyanka Shrestha, Bipin Adhikari

**Affiliations:** 1 Centre for Tropical Medicine and Global Health, Nuffield Department of Medicine, University of Oxford, Oxford, UK; 2 International Diagnostics Centre, London School of Hygiene & Tropical Medicine, London, UK; 3 Mahidol-Oxford Tropical Medicine Research Unit, Faculty of Tropical Medicine, Mahidol University, Bangkok, Thailand

**Keywords:** Antimicrobial resistance, Food animals, Human health, One Health

## Abstract

The use of antimicrobials in animals for growth promotion and infection prevention significantly contributes to the development of antimicrobial resistance (AMR), a growing public health threat. While the World Health Organization (WHO), the United Nations (UN) and the European Union (EU) have taken steps towards reducing and restricting the use of antimicrobials in animals, initiatives are insufficient in developing countries where the demands for food animals continue to rise over the years. The inter-sectoral acknowledgment of inextricable link between animal health, human health and the environment (One Health approach) is critical. Concerted and collaborative efforts among all the stakeholders are essential to deal with this complex problem of resistance.

## Background

Antimicrobial resistance (AMR) is a global threat that causes 700,000 deaths annually. The burgeoning AMR is estimated to account for 10 million deaths and US$ 100 trillion economic loss every year by 2050 if no urgent actions are taken [1]. The current approaches at tackling AMR suffer from inadequate multi-sectoral and cross-disciplinary efforts and thus demands urgent and concerted actions embedded in the ‘One Health approach’ [2]. One Health approach embraces efforts to redress the inappropriate use of antimicrobials in humans, food animals and environment.

Antimicrobials are widely used in animals to treat infections, prevent diseases and promote growth [3]. An estimated 131,109 tons of all antimicrobials were used in food animals in 2013 and the figure is projected to rise to 200,235 tons by 2030 [4]. A considerable variation on consumption between countries is reported; ranging from 8 mg/population correction unit (PCU) in Norway to alarmingly high (318 mg/PCU) in China [4]. Although, the use of antimicrobials is imperative in treating animals, most of its use is directed to prevent infections and promote growth [3,5,6]. In the United States, around 70% antimicrobials, which are used to treat human infections are sold for use in food animals [5]. Data available from 30 different countries in Europe exhibit a similar pattern [6]. Information from the developing countries is limited; however, empirical estimates suggest that the rampant use of antimicrobials in food animals is a serious concern [1,7]. Over the years, evidence has accrued to suggest that use of antimicrobials in animals has contributed to the development of AMR in humans. However, little has been focused on quantifying the burden and impact. In this commentary, we review relevant literature to scrutinize the current trend on the use of antimicrobials in food animals, its consequences and knowledge gaps, and explore possibilities for further research, policy and community engagement.

## Main text

### Antimicrobial use in food animals and antimicrobial resistance

The primary driver for evolution of resistant genes is the excessive use of antimicrobials that exerts selection pressure and thus escalates the favourable mutation in the bacteria [8]. Although antimicrobials are used in comparable amounts in animals and humans (118 mg/PCU and 133 mg/kg respectively), the chances of mutations in animals are higher due to larger animal biomass [4]. In addition, antimicrobials use in low (sub-therapeutic) dose in food animals as growth promoters allow a perfect environment for emergence of AMR. The level of antimicrobial resistance developed by the microbes in the animal population is positively correlated with the level of antimicrobial consumption in this population [9,10]. For example, high level of resistance in *E. coli* against medically important antibiotics such as penicillin, chloramphenicol, tetracycline, sulfonamides and fluroroquinolones have been attributed to overuse of these antimicrobials in poultry animals [10]. Repeated exposure to sub-therapeutic doses of antimicrobials also causes shifts in gut microbiomes, and promotes the growth of microbes and antimicrobial genes not only specific to the antimicrobial being used but also against other antimicrobials [11].

Food production industries in the developing countries, driven by increasing market demand and associated financial incentives, continue to use antimicrobials in growth promotion of food animals. Additionally, with improved economy and purchasing power in developing countries, per capita consumption of meat has substantially increased over previous decades, from 10.2 kg/year in 1964–66 to 25.5 kg/year in 1997–99 and is expected to rise to 36 kg/year by 2030 [12]. However, economic analysis using a large-scale empirical data in the United States, demonstrated that the use of growth promoters in the poultry production is associated with economic loss to the producers [13]. Rather than using antimicrobials as growth promoters in food animals, measures to prevent diseases with improved management have been found to be more effective in maintaining productivity [14].

### Impacts of environment

Environment serves as a significant transmission reservoir for many pathogens, perpetuating the cycle of consumption and contamination [15]. Pollution, poor sanitation, organic fertilizers and industrial wastes conduce to the reservoir of infections, contributing to the environmental resistome [16,17]. In addition, antimicrobials used as pesticides in developing world are exceeding the safety limits in edible crops. For instance, 93% of eggplant and 100% of tomato and chilli samples imported from India to Nepal contained residual antimicrobial pesticides [18]. Contamination of antimicrobials in soil and water ecosystems can lead to evolution of multi-resistant bacteria through metabolic processes [16]. Dissemination of antimicrobial resistance is reported in many *Salmonella* serotypes across commercial swine farms following manure applications [15].

The recent trade-offs with increasing urbanization and consequent compromise in ecological health in terms of sanitation and air quality [19] has stretched the public health system with high burden of infectious diseases. For instance, high air pollution in Delhi has led to an increased burden of respiratory infections, putting pressure on the demands of antimicrobials [20]. Increasingly, scientific reports have highlighted the role of environment in influencing the rise in AMR, however, surveillance systems and health policies have often failed to address the impact.

### Impacts on human health

Antimicrobial resistant bacteria originating in an animal can be transmitted to humans through the environment, food products, and/or by direct contact [8]. Similar strains of resistant bacteria are found in food animals and humans suggesting the bacterial transmission from animals to humans [21]. Plasmid-mediated resistance in *E. coli*, *Salmonella* and *Klebsiella* to colistin, a last line group of antimicrobial drug, is reported in both food animals and humans in countries from North America, Europe, Africa and Asia [17]. A recent systematic review demonstrated that a substantial proportion of human extra-intestinal extended spectrum cephalosporin-resistant *E. coli* infections originate from food animals [22]. Strong correlation has been found between the prevalence of amoxicillin resistance in *E. coli* isolated from food animals and humans (spearman correlation coefficient, r = 0.94) [23]. An intervention targeted to reduce the use of antimicrobials in animals showed 24% reduction in prevalence of resistant infections in humans [24].

Despite these evidence, some researchers argue that animals to human transfer of resistance is negligible, and even claim that restricting antimicrobials use in food animals may lead to detrimental effects on both animal and human health [25]. The polarized debate on the association and magnitude of effect on human health due to antimicrobial use in food animals demands more research exploring resistance dynamics and quantifying the effects on human and animal health [8].

### Current approaches

There is a growing consensus to stop the unnecessary and inappropriate use of antimicrobials in animals. Countries in the European Union (EU) have restricted the use of antimicrobials in disease prevention and growth promotion for several decades. The United Kingdom for the first time restricted authorization of several antimicrobials including tetracycline and penicillin based on the recommendation of the committee led by Professor Michael Swann in early 1969 [26]. Antimicrobial use as growth promoters was phased out from Sweden in 1986, Norway in 1995, and Denmark in late 1998–99 and was followed by all other EU countries, terminating the use of antimicrobials as growth promoters in 2006 [27].

The WHO Global Action Plan aims to optimize the use of antimicrobials in animal health, urging its member countries to develop National Action Plans to tackle AMR incorporating considerations of antimicrobial use in food animals [28]. WHO recommends reducing and restricting medically important antimicrobials in food animals for disease prevention and growth promotion [29]. Echoing with the WHO recommendations, the UN FAO action plan on AMR focuses on surveillance and monitoring, strengthening governance and promoting good practices of optimal use of antimicrobials in food and agriculture [30]. The World Organisation for Animal Health (OIE) share a similar commitment and collaborates with the WHO and the UN FAO to address these challenges [31]. Use of antimicrobials in animals has also gained a global attention during the United Nations General Assembly in September 2016. Need for investments in AMR has been recognized globally, for example, the UK government has established Fleming Fund to respond to the global threat of AMR which focuses on LMICs. Specifically, Fleming Fund aims to improve the surveillance of AMR through cross-disciplinary collaboration (One Health approach) to generate robust evidence for policy translation and program implementation [32]. Nonetheless, actions towards ‘One Health’ have not progressed as expected and still remains a global challenge.

A report by Organization for Economic Co-operation and Development (OECD) on agricultural policies and market states that only 42 countries have placed a system to collect and report the use of antimicrobials in food animals [14]. Efforts to compare the larger surveillance data between animals and humans are limited by the heterogeneity in sampling techniques, lab infrastructure, and reporting modalities across the countries [33]. Despite the commitment at the international level, policies and priorities vary between countries and regions. Efforts to implement the relevant recommendations is lagging and the consumption of antimicrobials in food animals continues to escalate over the years [34]. Although antimicrobials are strictly sold on prescriptions in the developed world, antimicrobials are substantially cheaper and readily available over the counter (without prescriptions) in developing countries [35], thus conducing its use for growth promotion and infection prevention in animals [8].

Countries in EU/EAA have taken steps towards standardization of AMR surveillance and measurement of antimicrobial consumption to evaluate progress made in reducing antimicrobial use in both human and animals [36,37]. However, measurement and surveillance of AMR incorporating the ‘One Health’ approach is constrained by lack of resources such as the technical capacity, previous experience of using One Health approach and funding in most of the developing countries [38]. High inappropriate use of antimicrobial is reported from the developing world and has been contributed by either ambiguous policy or inadequate implementation of existing policy on the use of antimicrobials among other various factors [2,35,39].

### Way forward

Robust surveillance systems that collaborate across all the sectors where antimicrobials are used are critical in generating evidence for policy change and implementation [38]. Research on alternative and more organic ways of improving animal health and agriculture, and enhancing food products should be encouraged. A national level policy discourse with stringent policy and regulatory system together with community engagement are urgently required.

## Conclusion

Future studies should invest in producing robust evidence on the “One Health” approach through a multi-sectoral and multidisciplinary partnership. A global initiative to devise a standard, cross-culturally valid tool to assess the surveillance data on antimicrobial use in animals and its impact on human health is critical. Policymakers, regulatory bodies, animal health practitioners, food producers, pharmaceutical companies and drug dispensers should prioritize inter-sectoral collaboration to restrict inappropriate antimicrobials use in animals.

## Data Availability

Not applicable.
